# Application of a Deep Learning System in Pterygium Grading and Further Prediction of Recurrence with Slit Lamp Photographs

**DOI:** 10.3390/diagnostics12040888

**Published:** 2022-04-02

**Authors:** Kuo-Hsuan Hung, Chihung Lin, Jinsheng Roan, Chang-Fu Kuo, Ching-Hsi Hsiao, Hsin-Yuan Tan, Hung-Chi Chen, David Hui-Kang Ma, Lung-Kun Yeh, Oscar Kuang-Sheng Lee

**Affiliations:** 1 Department of Ophthalmology, Chang-Gung Memorial Hospital, Linkou, No. 5, Fu-Hsing St., Kuei Shan Hsiang, Taoyuan 83301, Taiwan; agarlic2000@gmail.com (K.-H.H.); hsiao.chinghsi@gmail.com (C.-H.H.); tanhsin@gmail.com (H.-Y.T.); mr3756@cgmh.org.tw (H.-C.C.); davidhkma@yahoo.com (D.H.-K.M.); 2 College of Medicine, Chang-Gung University, No. 259 Wen-Hwa 1st Road, Kuei Shan Hsiang, Taoyuan 33302, Taiwan; zandis@gmail.com; 3 Institute of Clinical Medicine, National Yang Ming Chiao Tung University, No. 201, Sec.2, Shih-Pai Rd. Peitou, Taipei 11221, Taiwan; 4 Center for Artificial Intelligence in Medicine, Chang-Gung Memorial Hospital, Taoyuan 83301, Taiwan; lin3031@gmail.com; 5 Department of Electronic Engineering, Chang-Gung University, Guishan District, Taoyuan 33302, Taiwan; 6 Department of Information Management, National Chung Cheng University, Chiayi 62102, Taiwan; bmajsr@ccu.edu.tw; 7 Division of Rheumatology, Allergy and Immunology, Chang-Gung Memorial Hospital, Taoyuan 83301, Taiwan; 8 Stem Cell Research Center, National Yang Ming Chiao Tung University, Taipei 11221, Taiwan; 9 Department of Orthopedics, China Medical University Hospital, Taichung 40447, Taiwan

**Keywords:** automatic pterygium grading, deep learning system, slit-lamp photograph, prediction of pterygium recurrence

## Abstract

Background: The aim of this study was to evaluate the efficacy of a deep learning system in pterygium grading and recurrence prediction. Methods: This was a single center, retrospective study. Slit-lamp photographs, from patients with or without pterygium, were collected to develop an algorithm. Demographic data, including age, gender, laterality, grading, and pterygium area, recurrence, and surgical methods were recorded. Complex ocular surface diseases and pseudopterygium were excluded. Performance of the algorithm was evaluated by sensitivity, specificity, F1 score, accuracy, and area under the receiver operating characteristic curve. Confusion matrices and heatmaps were created to help explain the results. Results: A total of 237 eyes were enrolled, of which 176 eyes had pterygium and 61 were non-pterygium eyes. The training set and testing set were comprised of 189 and 48 photographs, respectively. In pterygium grading, sensitivity, specificity, F1 score, and accuracy were 80% to 91.67%, 91.67% to 100%, 81.82% to 94.34%, and 86.67% to 91.67%, respectively. In the prediction model, our results showed sensitivity, specificity, positive predictive value, and negative predictive values were 66.67%, 81.82%, 33.33%, and 94.74%, respectively. Conclusions: Deep learning systems can be useful in pterygium grading based on slit lamp photographs. When clinical parameters involved in the prediction of pterygium recurrence were included, the algorithm showed higher specificity and negative predictive value in prediction.

## 1. Introduction

Pterygium is a progressive disease with wing-shaped, fibrovascular tissue, mostly encroaching on the cornea from nasal bulbar conjunctiva. The etiology of pterygium is still under debate; however, age, gender, ultraviolet light exposure [1], virus infection [2], ocular demodicosis [3], and socioeconomic status [1] are considered as predisposing risk factors. Surgical excision is required in management of advanced pterygium, including simple excision with bare sclera, excision combined with antimetabolic agents (5-fluorourasil, mitomycin), amniotic membrane transplant (AMT), and auto-conjunctival graft. 

Different grading systems of pterygium severity have been developed for clinical purposes. The translucency-based grading system proposed by Tan and his colleagues was used to assess the recurrence of pterygium after bare sclera excision and conjunctival autografting [4]. Another grading system, with numerous parameters including Stocker’s line, the size and body length of pterygium, conjunctival injection, and thickness of the lesion, was later considered an assessment tool for pterygium surgery [5]. Liu et al. [6]. proposed the use of pre-operative caruncle gradings to estimate the outcome of multi-recurrent pterygium surgery, including recurrence and post-operative diplopia. They found that grading was strongly associated with post-operative diplopia, success rate, and residual conjunctiva. By contrast, Huang and his coworkers chose slit-lamp photographs as a simple method of pterygium grading. The advantage of this system is its easy application in clinical settings [7]. Instead of slit-lamp photographs, the topography-based grading system provides another way to evaluate the severity of pterygium. However, availability of this armamentarium, along with diverse manufacturers with various designs in corneal topography, limit its clinical application [8]. All the above-mentioned methods mainly focus on pre-operative assessment of pterygium severity; some other strategies use post-operative phenotypes to predict surgical outcome by appearance of the ocular surface [8,9].

When developing an efficient and automatic grading system for pterygium, we can take artificial intelligence (A.I.) into consideration as one of the solutions. Machine learning techniques have been utilized to study eye disease in the cornea and retina [10]. Image processing algorithms, through established automatic systems, special segmentation techniques, or identifying features of redness, have been suggested for use in pterygium detection [11]. Since slit-lamp photography has its own merits, including easy access and high reproducibility, it can be used as an ideal source of images to create an algorithm of image-based grading system for pterygium. In this study, we developed a deep learning system (DLS) to perform pterygium grading, and further predict the surgical prognosis, in combination with other clinical information. 

## 2. Materials and Methods

### 2.1. Patients

One hundred and forty one patients (258 eyes) were surveyed in this retrospective, cross-sectional study, including patients with primary and recurrent pterygium, as well as those without pterygium. The research was approved by the Institutional Review Board of Chang Gung Memorial Hospital, Linkou (No. 202101418B0C501), and adhered to the tenets of the Declaration of Helsinki. Diagnosis of primary and recurrent pterygium was based on clinical examination, slit-lamp photography, and patients’ medical history. Frontal and lateral slit-lamp photographs of the ocular surface were taken and classified according to the published grading system for pterygium by single ophthalmologist (K.H.H) [7]. In brief, the grading system for pterygium severity in this study was based on the color and visibility of pterygium vasculature in the limbal area. 

### 2.2. Deep Learning System (DLS)

**Figure 1:**
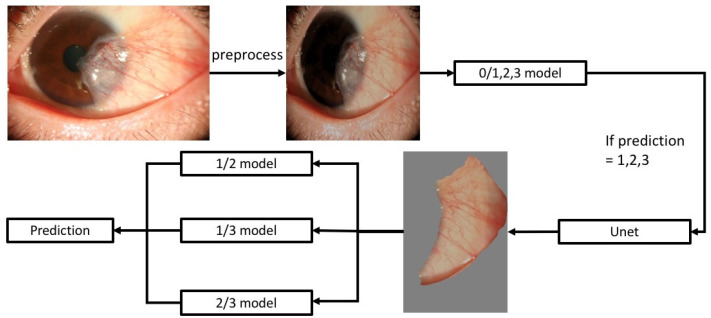
Flow chart of the development of deep learning system for autonomic pterygium grading. Following image augmentation and processing, pterygium was first identified, and then segmented with Unet. Further comparison was performed for severity grading.

After reviewing images of pterygium and non-pterygium, we excluded images lacking enough quality or medical history. All data were anonymous and unidentifiable for the developers of the DLS. Augmentation of the slit-lamp photographs was performed first, to increase the number and variability of images. In our algorithm, pre-processed pictures were first trained with a training set to confirm whether pterygium existed or not. Then, images of confirmed pterygium were further segmented [12], compared, and classified among different severities to show their final grading. Flow chart of our study design was shown in Figure 1. Sensitivity, specificity, accuracy, and F1 score were retrieved and presented with a confusion matrix and heat map.

### 2.3. Data Process and Modeling

We built three different AI models. The first model aimed to classify the presence of pterygium, while the second aimed to segment the sclera part from the rest of the image. Results from the first and second models were fed to the third model, which consisted of three subunits (classification of category 1 vs. 2; 2 vs. 3; 1 vs. 3; Figure 1). Finally, the results from model 3 were produced by voting. The design of our model referred to published articles [13,14]. 

**Figure 2:**
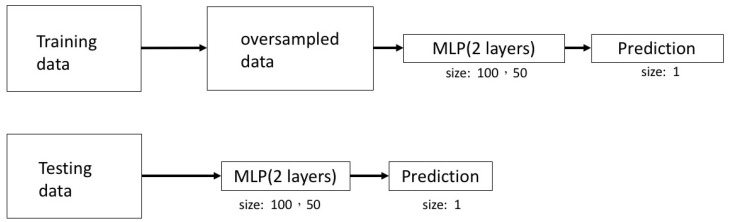
Structures of deep learning system for prediction of pterygium recurrence. In the training set, data were oversampled, followed by the multilayer perceptron (MLP) and prediction. In the testing data, images were processed through the MLP to predict pterygium recurrence.

In the algorithm for prediction of pterygium recurrence, patients and images were first grouped into recurrence and non-recurrence subgroup, and then trained with a training set (train:validation set = 2:1). Data was first oversampled to improve signal-to-noise ratio in training set, followed by processing in layers to predict pterygium recurrence (Figure 2). Demographic data, such as age, gender, laterality, grading, type of operation, and area of pterygium in clock hours were included as clinical parameters to predict recurrence after pterygium excision. 

## 3. Results

One hundred and forty one patients were reviewed in this study. After excluding images without enough quality or clinical data, 134 patients (237 eyes) were enrolled, including 73 pterygium patients and 61 patients without pterygium. Of the patients, 71 were male and the rest, 63, were female. The mean age of enrolled patients was 57.91 ± 14.81 years. A total of 176 eyes presented images of pterygium, compared to 61 eyes without pterygium. During the development of our algorithm, all images (237 eyes) were first trained in the DLS, for the algorithm to learn to make a diagnosis of pterygium, between grade 0 and other severities, followed by training between different pterygium gradings, to increase performance. Since there was no grade 4 pterygium in our database, we focused our grading algorithm on grades 0 to 3. Details of the image distribution between different gradings is summarized in Table 1.

**Figure 3:**
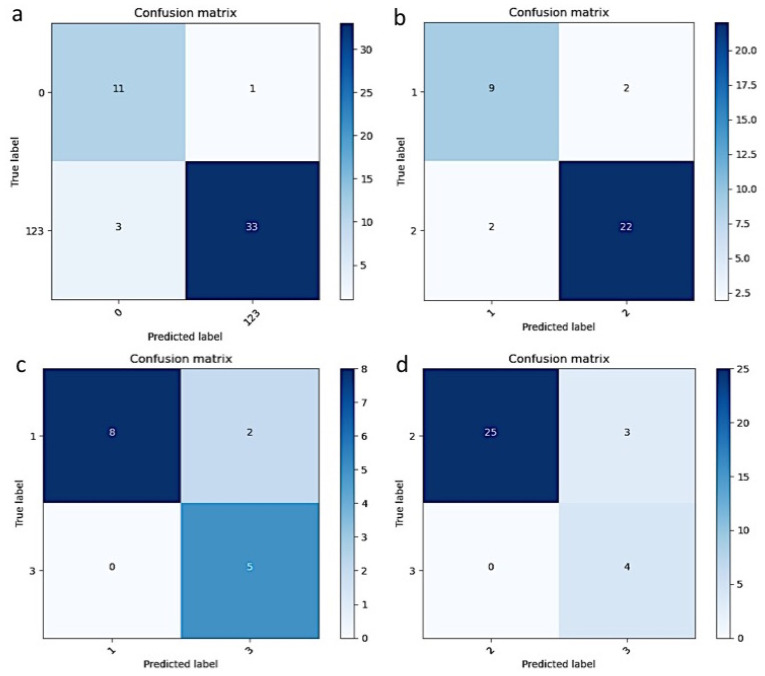
Confusion matrix of automatic pterygium grading. The results of pterygium screening in 48 cases showed three pterygia were misclassified as normal (**a**). Different severities of pterygium were further compared with each other: grade 1 & 2 (**b**), grades 1 & 3 (**c**), and grades 2 & 3 (**d**).

**Figure 4:**
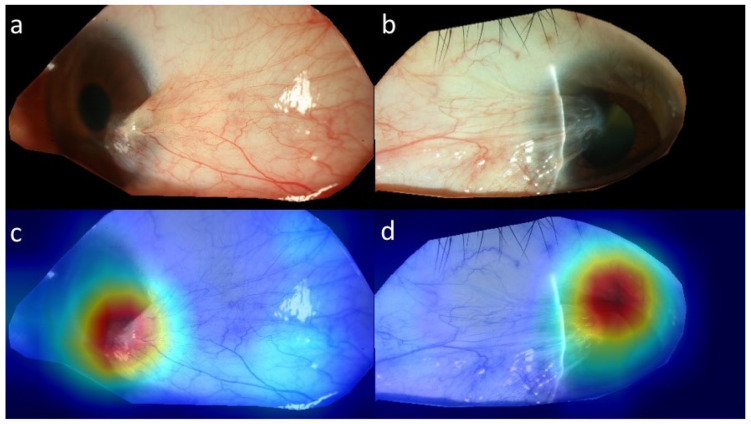
The results of images after cropping, with their corresponding heatmaps. Pterygium in the right eye (**a**) and left eye (**b**) after cropping. Heatmaps show the weighted area at the head of pterygium in the right (**c**) and left eye (**d**).

To separate non-pterygium from pterygium, our result showed that sensitivity, specificity, accuracy, and F1 score were 0.917, 0.917, 0.917, and 0.846, respectively. The confusion matrix showed that only 4 out of 48 were not correctly classified, of which three images of pterygium were misinterpreted as a normal, or unaffected, eye (Figure 3a). To automatically perform pterygium grading, different subgroups in the algorithm showed sensitivity between 80% and 89.29%, specificity from 91.67% to 100%, F1 score from 81.82% to 94.34%, and accuracy between 86.67% and 90.63%. Details of the outcome measures in different subgroups are shown in Table 2. The confusion matrix between different subgroups is shown in Figure 3b–d. In our results, pterygium images tended to be classified into a more severe grading by the DLS. Figure 4 showed images, after cropping to reduce noise, of the right eye (Figure 4a) and left eye (Figure 4b). Heatmaps present the highlighted area at the head of pterygium (Figure 4c,d). 

**Figure 5:**
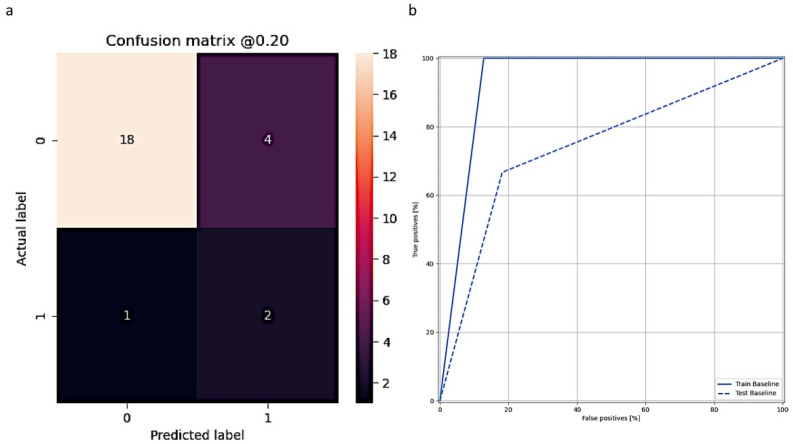
Confusion matrix and AUC curve in the prediction model for pterygium recurrence. In predicting pterygium recurrence, some patients (4 in 22) without recurrence were misinterpreted as having recurrent potential (**a**). AUC curve shows results of the train and test sets (**b**).

After verifying the algorithm for pterygium grading, we further combined clinical parameters with our grading result to predict pterygium recurrence in patients receiving pterygium excision. In our database, no recurrence was recorded in 108 cases, compared to fifteen cases with recurrent history. Of 108 patients, 86 cases were designated for the training set, and the rest (22 cases) were dispatched to the test set. Of fifteen recurrent patients, twelve cases were sent to the training set and three cases were dispatched to the test set. In our algorithm for prediction of recurrence, the results showed sensitivity, specificity, positive predictive value (PPV), negative predictive value (NPV), and accuracy were 66.67%, 81.82%, 33.33%, 94.74%, and 80%, respectively. Details of the outcome measures of the prediction model are shown in Table 3. The confusion matrix and AUC curve of our prediction model are presented in Figure 5. In the confusion matrix, four patients, who may not develop pterygium recurrence, were predicted to recur potentially. By contrast, only one recurrent case was incorrectly predicted to maintain no recurrence after pterygium excision. 

## 4. Discussion

In this study, we developed a DLS to automatically perform pterygium grading, simply based on both frontal and lateral slit-lamp photographs. After retrieving automatic grading through the DLS, we further verified an algorithm, combined with clinical parameters including age, gender, surgery type, and area of pterygium, to predict pterygium recurrence after excision. Only 237 eyes were enrolled in the final study, with at least two images of the frontal and lateral view of each eye, combined with image augmentation to help increase the number and variety of images for DLS development. Our results showed high specificity, from 91.67% to 100%, and sensitivity from 80% to 91.67% in pterygium grading. In the advanced application, our model for the prediction of pterygium recurrence showed the results of sensitivity, specificity, and accuracy were 66.67%, 81.82%, and 80%, respectively. 

Since pterygium is viewed as a proliferative disease with a strong potential for recurrence, pterygium grading and predisposing factors of recurrence are both key issues that concern researchers. Several grading systems have been proposed to classify pterygium into different severities [4,5,6,7,8], of which some systems required specific facilities, such as corneal topography and OCT, to measure the clinical parameters of pterygium. Instead, frontal and lateral view slit lamp photographs were chosen, in our study, to easily access clinical images of pterygium, according to the published method by Huang et al. [7]. The advantage of our model is its potential to be widely applied in slit-lamp photography-equipped medical care, and further combined with other clinical information to predict surgical outcome and visual prognosis. A slit-lamp photography-based grading system requires clear images of the frontal and lateral view of the pterygium, including especially detailed vasculature and transparency of the corneo-limbal area in the lateral view. Therefore, a limbal area-centered cropping process was equipped in our model before grading to improve the identification of subtle difference between grades (Figure 1); however, this management also led to loss of image information outside the cropped area, at the same time. The disadvantage of this simple grading method resulted from the absence of biomedical information of pterygium, such as tissue thickness, size, and its cellular components. Meanwhile, quality of images could also affect performance of grading in our algorithm. 

Most DLSs in published articles were developed for the automatic diagnosis of pterygium, rather than advanced grading [15,16]. Xu and his associates reported that sensitivity, specificity, and F1 scores in their results were 90.06 to 100%, 95.56 to 99.64%, and 89.47 to 99.74% in patient groups of normal, pterygium under observation, and pterygium receiving excision, respectively [15]. In their study design, only one slit-lamp photograph was selected from each person, and the diagnosis was made between these three groups without a specific grading system. Furthermore, Fang and his colleagues presented AUC more than 99% to detect pterygium in their internal and external test set, and AUC from 98.5 to 99% in referable pterygium [16], which was also verified in hand-held photographs for telemedicine potential. Although several grading systems for pterygium have been proposed, no conclusion has yet been made about which one is most clinically applicable. Therefore, it is worthy to explore how to use clinically available facilities to perform automatic grading.

In our results, a higher specificity than sensitivity was found, which may be attributed to the characteristics of pterygium and the criteria of the grading system. Clinically, pterygium encroaching onto the cornea is rarely misinterpreted as normal ocular surface, but differential diagnosis is required to identify other conjunctival diseases, such as conjunctival cyst, phlyctenulosis, and pseudopterygium. In one published article, a DLS was applied to differentiate pterygium from other eye diseases, such as keratitis, subconjunctival hemorrhage, and cataract [17]. However, comparison between different severities of pterygium was still lacking. In the real world, conjunctival transparency and vasculature vary at the limbal area across a wide range, and are interwoven in 3D structure; therefore, definition of obscured deep episcleral and scleral vessels and conjunctival redness may be not easily interpreted by DLS. The above-mentioned reason may explain variation of sensitivity in our results. Furthermore, few grade 4 images were included in our database, according to the original criteria of pterygium grading [7]. To solve this problem, we can perform pterygium grading first through the developed algorithm, and then locate outsiders with grade 4 pterygium as a flexible strategy, or consider using an external database with more grade 4 images to verify the algorithm. In sum, an automatic grading system for pterygium could provide a quick report for ophthalmologists and help them explain diseases severity to patients and make an evidence-based referral for a surgical management. Moreover, for general physicians, an automatic grading system could reduce unnecessary consultations for pterygium.

Prediction of pterygium recurrence is still a main issue before surgical excision. Age, race, surgical type, use of mitomycin C, dry eyes, latitude, and pterygium morphology have been proposed as potential environmental and surgical factors of recurrence [18,19,20,21]. Accordingly, age, gender, grading, surgical procedure, area of pterygium base (clock hours), and laterality were enrolled in our prediction model for the estimation of recurrence after pterygium excision. Our model showed higher specificity (86.11%) and NPV (98.93%) than sensitivity (51.72%) and PPV (48.27%), which means that more patients without recurrence potential will be misinterpreted as likely to recur after surgery, and most patients predicted not to recur are reliable. Our results showed that it may be feasible to develop a customized prediction model, according to clinical demographics, surgeon’s personal technique, size of lesion, and an automatic grading system. With the assistance of a prediction model, ophthalmologists could be quickly advised to make a decision to address varieties of pterygium. In clinical practice, this model helps health providers to choose a better strategy to reduce recurrence in pterygium patients beforehand; however, overtreatment and more medical costs are major disadvantages. Furthermore, this is a cross-sectional study, in which patients without recurrence currently may develop recurrent pterygium in the future.

In predicting recurrence, different factors can be added into the algorithm to estimate surgical outcome. Although we chose a different grading system than those in published articles, the parameters of other grading systems can still be combined with the results of automatic grading in developing an algorithm for the prediction of recurrence, if associated facilities are available in the health care system. In our future work, reports of automatic grading could be combined with other clinical parameters, before and after surgery, to become an accurate prediction tool in surgery, which could help decision making and outcome prediction. There were some limitations in our study, including a low variety of ocular surface diseases in our images, few grade 4 pterygium enrolled, relatively homogenous ethnic background of participants, few recurrent cases in our database, and no verification of our algorithm with an external database. General application of our model to hand-held photography systems, such as a smart phone or portable camera, would be a future challenge.

## Figures and Tables

**Table 1:** Images dispatched for training and testing group in various strategies.

Classification (Grading)	Training Set	Test Set
0 and 1, 2, 3	189	48
1 and 2	104	35
1 and 3	59	15
2 and 3	75	32

**Table 2:** Outcome measures of the deep learning system in different pterygium gradings.

Classification (Grading)	Sensitivity	Specificity	F1 Score	Accuracy
0 and 1, 2, 3	0.9167	0.9167	0.8462	0.9167
1 and 2	0.8182	0.9167	0.8182	0.8857
2 and 3	0.8929	1.0000	0.9434	0.9063
1 and 3	0.8000	1.0000	0.8889	0.8667

**Table 3:** Performance of deep learning system in predicting pterygium recurrence.

Statistics	Value	95% Confidence Interval (CI)
sensitivity	66.67%	9.43–99.16%
specificity	81.82%	59.72–94.81%
PPV	33.33%	13.16–62.27%
NPV	94.74%	78.21–98.90%
accuracy	80.00%	59.30–93.17%

PPV, positive predictive value; NPV, negative predictive value.

## Data Availability

Not applicable.
